# Radiological and Pathological Features Associated with IDH1-R132H Mutation Status and Early Mortality in Newly Diagnosed Anaplastic Astrocytic Tumours

**DOI:** 10.1371/journal.pone.0123890

**Published:** 2015-04-07

**Authors:** Jason K. Wasserman, Garth Nicholas, Rebecca Yaworski, Anne-Marie Wasserman, John M. Woulfe, Gerard H. Jansen, Santanu Chakraborty, Thanh B. Nguyen

**Affiliations:** 1 Division of Laboratory Medicine, Department of Anatomical Pathology, Ottawa, Ontario, Canada; 2 The Ottawa Hospital Cancer Centre, Ottawa, Ontario, Canada; 3 Division of Neuro-imaging, Department of Medical Imaging, Ottawa, Ontario, Canada; 4 University of Ottawa, Ottawa, Ontario, Canada; 5 The Ottawa Hospital Research Institute, Ottawa, Ontario, Canada; Baylor College of Medicine, UNITED STATES

## Abstract

**Background:**

Glioblastoma can occur either de novo or by the transformation of a low grade tumour; the majority of which harbor a mutation in isocitrate dehydrogenase (IDH1). Anaplastic tumours are high-grade gliomas that may represent the final step in the evolution of a secondary glioblastoma or the initial presentation of an early primary glioblastoma. We sought to determine whether pathological and/or radiological variables exist that can reliably distinguish IDH1-R132H-positive from IDH1-R132H-negative tumours and to identify variables associated with early mortality.

**Methods:**

Patients diagnosed with anaplastic astrocytic tumours were included. Magnetic resonance imaging was performed and immunohistochemistry was used to identify tumours with the IDH1-R132H mutation. Survival was assessed 12 months after diagnosis. Variables associated with IDH1-R132H status were identified by univariate and ROC analysis.

**Results:**

37 gliomas were studied; 18 were positive for the IDH1-R132H mutation. No tumours demonstrated a combined loss of chromosomes 1p/19q. Patients with IDH1-R132H-positive tumours were less likely to die within 12 months of diagnosis (17% vs. 47%; p=0.046), more likely to have tumours located in the frontal lobe (55% vs. 16%; p=0.015), and have a higher minimum apparent diffusion coefficient (1.115 x 10^-3^ mm^2^/sec vs. 0.838 x 10^-3^ mm^2^/sec; p=0.016), however, these variables demonstrated only moderate strength for predicting the IDH1-R132H mutation status (AUC=0.735 and 0.711, respectively). The Ki-67 index was significantly lower in IDH1-R132H-positive tumours (0.13 vs. 0.21; p=0.034). An increased risk of death was associated with contrast-enhancement ≥ 5 cm^3^ in patients with IDH1-R132H-positive tumours while edema ≥ 1 cm beyond the tumour margin and < 5 mitoses/mm^2^ were associated with an increased risk of death in patients with IDH1-R132H-negative tumours.

**Conclusions:**

IDH1-R132H-positive and -negative anaplastic tumours demonstrate unique features. Factors associated with early mortality are also dependent on IDH1-R132H status and can be used to identify patients at high risk for death.

## Introduction

Glioblastoma is the most common type of primary malignant brain tumour in adults. Despite optimal medical and surgical management, the majority of patients die from the disease within 12–16 months of diagnosis.[1] According to the current WHO classification, astrocytic tumours that have an inherent tendency to progress to glioblastoma are defined as diffuse (WHO grade II) or anaplastic (WHO grade III) while glioblastoma is given the highest grade (WHO grade IV).[2] Glioblastoma can either occur de novo ('primary glioblastoma') or it can develop from a previously diagnosed lower grade tumour ('secondary glioblastoma'); the majority of tumours are primary glioblastomas.[3] Important recent discoveries have shown that most secondary glioblastomas arise from lower grade astrocytic tumours that harbor a mutation in the gene for isocitrate dehydrogenase (IDH1); of these, 90% posses the R132H mutation.[4] In contrast, the mutation is absent in the great majority of primary glioblastomas.[4] Anaplastic astrocytic tumours (anaplastic astrocytoma [AA] and anaplastic oligoastrocytoma [AOA]) exist at the interface between these two entities with just over half of tumours harboring an IDH1 mutation. As such, an anaplastic glioma may represent progression of a low-grade glioma into a high-grade lesion or the initial presentation of an early primary glioblastoma. How these tumours differ in their biological behavior at this critical time point in their development still remains poorly understood.

Magnetic resonance imaging (MRI) plays a critical role in both the diagnosis and management of patients with glioma. Radiological features that are used to characterize a glioma include the size, shape, and location of the tumour, contrast enhancement (CE), the presence of peri-tumoural edema, apparent diffusion coefficient and mass effect. Recent evidence has shown that IDH1 mutant diffuse gliomas and glioblastomas demonstrate unique radiological features that correlated with outcome.[5, 6] Specifically, a study examining diffuse gliomas demonstrated that IDH1 mutant tumours were smaller and less infiltrative.[6] In contrast, a study examining glioblastoma found that IDH1 mutant tumours were more likely to be non-CE, larger, and in the frontal lobe.[5] To date there have been no similar studies focusing on patients diagnosed with AA despite the wide variability in outcome among patients with this tumour. Knowledge of IDH1 status in the context of specific radiological criteria would allow clinicians to provide patients with a more accurate prognosis and in the future may better predict their response to targeted therapies.

The present study assessed a defined set of MRI variables and two markers of cellular proliferation in patients presenting with newly diagnosed anaplastic astrocytic tumours (AA and OAA) in order to better understand their biological evolution. This patient population was selected because diffuse astrocytic tumours (grades II and III) invariably transform into glioblastoma; AA and AOA are entities at a crucial transition point. The purpose of this study was to determine whether any of the radiological or pathological variables assessed could reliably distinguish IDH1-R132H positive from IDH1-R132H negative tumours in this patient population. We also sought to identify radiological and pathological variables associated with early mortality in order to provide clinicians with better prognostic tools for managing this uncommon yet challenging patient population.

## Methods

### Study population

This study was approved by the Ottawa Hospital Research Ethics Board (20130596-01H). Patient consent was not obtained for this retrospective study however all data were analysed anonymously and no patient identifiers exist in the manuscript. The database of the Department of Anatomical Pathology was used to identify all patients with a histological diagnosis of a WHO grade III anaplastic astrocytic tumour, either AA or AOA, between January 2010 and May 2013. Patients with pure anaplastic oligodendrogliomas were not included because these tumours are not traditionally considered a precursor to glioblastoma. Patients with a previous diagnosis of a low-grade (WHO grade II) brain tumour or insufficient tissue for immunohistochemical analysis were excluded.

### Clinical variables

The following clinical variables were collected: age, gender, and the presence of weakness, sensory changes, seizure, headache, visual disturbance, or dizziness at presentation. All patients underwent either a stereotactic biopsy or surgical resection.

### Treatment

Treatment consisted of temozolomide with or without concomitant radiation therapy. Temozolomide was administered according to the Stupp protocol.[7] Patients received between 40 and 60 Gy of external beam radiation in 30 fractions. For three patients, radiation was administered by Cyberknife.

### Pathological assessment

All histological sections were reviewed by two neuropathologists according to the 2007 WHO classification of CNS tumours. Mitotic index was calculated by counting mitotic figures in 50 high-power fields with the final index expressed as mitoses per mm^2^. Fields were identified on low-power based on high cellularity and lack of necrosis. Cell proliferation was assessed by immunohistochemistry for Ki67 (clone MIB-1, 1:100; Dako) and the Ki67 labelling index (LI) was assessed using the 'hot-spot' method. Using this method, areas of high Ki67 labelling were identified at scanning magnification and 1000 positively labelled tumour cells were subsequently counted in four high-power fields. The Ki67 LI was then calculated as the number of Ki67-positive cells divided by the total number of tumour cells in each field.

### Assessment of IDH1 mutation status and loss of 1p/19q

IDH1 mutation status was assessed by immunohistochemistry using an antibody specific for the R132H mutation (clone H09, 1:100 dilution; Dianova). Consequently, for the purpose of this study, the designation of a tumour as 'IDH1 positive' refers only to the R132H mutation. A cell was considered positive if it demonstrated dark cytoplasmic staining with or without dark nuclear staining. This method has been shown to have high sensitivity and specificity when compared to polymerase chain reaction.[8]

The status of chromosomes 1p and 19q was assessed by fluorescence in situ hybridization (FISH) on paraffin-embedded tissue with human probes localizing to 1p32, 1q25, 19q13, and 19p13. The test was conducted by counting the number of probe signals within 40 tumour cells for each of the two probes and calculating the average probe number per cell. Tumours containing a 1p36/1q25 ratio less than 0.88 and a 19q13/19p13 ratio less than 0.74 were considered to be deleted for 1p36 and 19q13, respectively.

### Magnetic resonance imaging

All patients received a MRI as part of the routine work-up for newly diagnosed brain tumours. Standard axial T1 pre- and post contrast, axial FLAIR, axial T2, coronal T1 post contrast images, axial diffusion-weighted imaging (b = 0/500/1000) were obtained from the brain on a 1.5T or 3T clinical scanner before and after surgery. A neuroradiologist blinded to the IDH status of the tumour assessed the preoperative MR exam for specific pre-defined features (Table 1).

**Table 1:** Features and associated descriptors assessed on the pre-operative MR exam.

Feature	Descriptor
*Tumour location*	Frontal
	Non-frontal
*Tumour volume*	Measured in cm^3^
*Contrast-enhancing tumour volume*	Measured in cm^3^
*Degree of enhancement*	Less than the choroid plexus
	Equal to/greater than the choroid plexus
*Heterogeneity of enhancement*	No enhancement/homogenous enhancement
	Heterogeneous enhancement
*Peri-tumoural edema*	Absent
	< 1 cm from tumour margin
	≥ 1 cm from tumour margin
*Mass effect*	None/mild
	Moderate/severe
*Intra-tumoural hemorrhage*	Present
	Absent
*Cyst formation*	Present
	Absent
*Tumour margin*	Irregular
	Smooth
*Minimum apparent diffusion coefficient*	Measured in mm^2^/sec

The maximal axial diameters and the vertical height of the entire tumour and of its enhancing component were measured on T1 post contrast or/and FLAIR images. Total tumour volume and enhancing-tumour volume were calculated according to the following formula: axial diameter x vertical height x 0.52. From visual inspection of the diffusion-weighted images, a small region-of-interest (area = 25 mm^2^) was placed in the area of lowest apparent diffusion of coefficient (ADC) in the tumour.

Type of surgery (biopsy, gross total resection or partial resection) was determined from operative reports and was confirmed by post-operative MRI. In patients who underwent surgical resection, a gross total resection was defined as the absence of enhancing or non-enhancing tumour surrounding the surgical cavity on the post-operative MRI. Patients with any residual enhancing or non-enhancing tumour were classified as having a partial resection.

### Outcome

The primary outcome for this study was death within 12 months from the initial date of diagnosis. In our institution patients are followed by both their medical oncologist and radiation oncologist, as appropriate.

### Statistical analysis

The data are presented as means ± standard deviation (SD) for continuous variables and counts with percentages for categorical variables. Differences in continuous variables were analyzed using the Mann-Whitney U-test while categorical variables were evaluated using the Chi-squared test or Fisher's exact test as appropriate. Receiver operating characteristics (ROC) were used to assess the predictive power of each radiological feature for the IDH1 mutation status of the tumour. For all variables assessed, the ROC analysis was used to find the optimal cut point for both sensitivity and specificity and this cut point value was used in the subsequent analysis of variables associated with early mortality. All analyses were univariate; due to the small sample size, a multivariate analysis was not performed. Values with a p < 0.05 were considered statistically significant. SPSS 20.0 (SPSS for Windows; SPSS Inc., Chicago, IL, USA) was used for all statistical analysis.

## Results

### Association between clinical variables and IDH1-R132H mutation status

**Fig 1:**
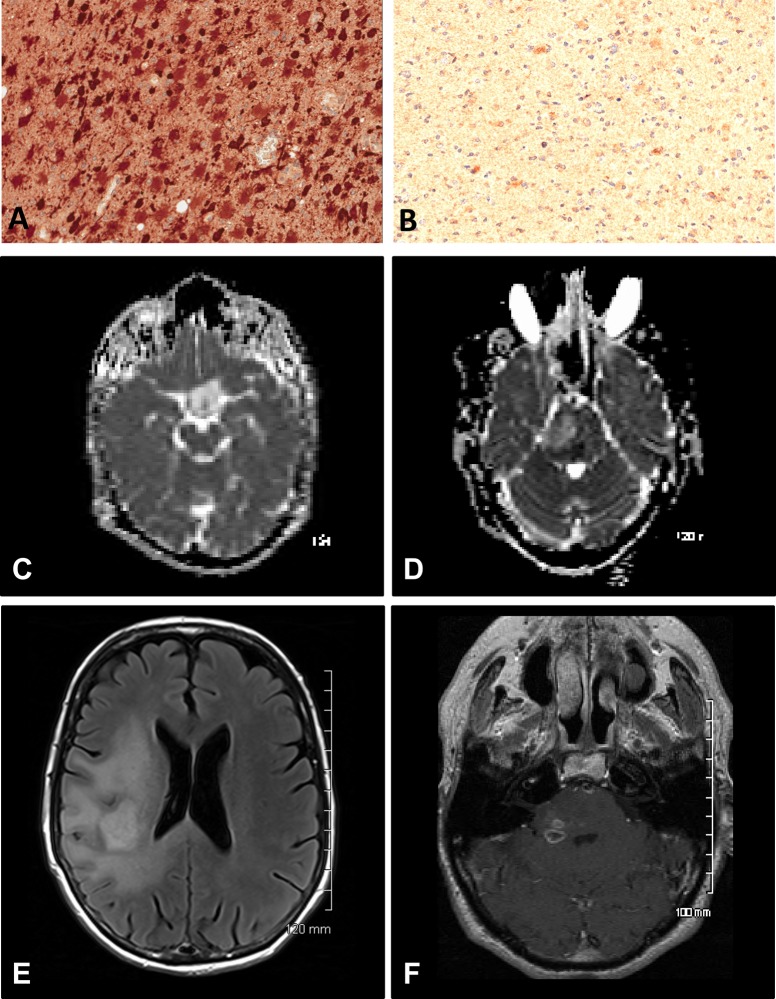
Pathological and radiological differences between IDH1-R132H positive and IDH1-R132H negative tumours. A and B: Immunohistochemical staining was used to identify IDH1-R132H positive tumours which demonstrated intense cytoplasmic staining (A) relative to the unstained tumour cells in IDH1-R132H negative tumours (B). C and D: An IDH1-R132H positive tumour (C) demonstrating a higher minimum ADC relative to an IDH1-R132H negative tumour (D). E and F: An IDH1-R132H positive tumour (E) demonstrating a greater volume of contrast-enhancing tumour relative to an IDH1-R132H negative tumour (F).

A total of 37 patients with newly diagnosed anaplastic astrocytic tumours were included in this study, including 28 AA and 9 AOA. The demographic, clinical, and molecular data for all the patients included in subsequent analyses are summarized in Table 2. Twelve patients (33%) died within 12 months of their initial diagnosis. The IDH1-R132H mutation was detected by immunohistochemistry in 18 tumours (49%) (Fig 1A and 1B). Anaplastic oligoastrocytomas accounted for 33% and 15% of the tumours in the IDH1-positive and IDH1-negative groups, respectively. All three tumours demonstrating a loss of 1p or 19q were also IDH1-R132H positive. No tumours demonstrated a combined loss of 1p/19q.

**Table 2:** Demographic, clinical, and molecular data for patients with newly diagnosed anaplastic astrocytic tumours.

	Number of patients	Percentage
*Gender*		
Male	16	43
Female	21	57
Age, mean (range)	48 (20–81)	-
*Symptoms*		
Weakness	15	41
Sensory	5	14
Seizure	16	43
Headache	10	27
Vision	3	8
Dizziness	3	8
*Surgery*		
Biopsy only	12	32
Partial resection	22	60
Gross total resection	3	8
*Adjuvant therapy*		
Yes	33	89
Chemotherapy	27	73
Radiation therapy	32	87
Chemo + radiation	25	68
*Outcome*		
Clinical progression	23	62
Death	12	32
*Histological subtype*		
Anaplastic astrocytoma	28	76
Anaplastic oligoastrocytoma	9	24
*IDH1-R132H mutation status*		
Positive	18	49
Negative	19	51
*1p/19q status*		
Loss of 1p	1	3
Loss of 19q	2	5
Loss of 1p and 19q	0	0

Patients with IDH1-R132H positive tumours however were significantly younger (44.0 vs. 54.0; p = 0.038). Indeed, 72% of patients with IDH1-R132H positive tumours were younger than 50 years old whereas 68% of patients with IDH1-R132H negative tumours were older than 50 years. Compared to patients over the age of 50 years, young patients were also more likely to present with seizure (63% vs. 22%; p = 0.02). Patients with IDH1-R132H positive tumours were less likely to die within 12 months of the initial diagnosis (17% vs. 47%; p = 0.046). There were no differences in clinical presentation, surgical or medical management between patients with IDH1-R132H positive and IDH1-R132H negative tumours.

### Association between MRI features and IDH1-R132H mutation status

All patients underwent MRI prior to surgery. Ten out of 18 IDH1-R132H positive tumours (55%) were located in the frontal lobe. In contrast, only 3 of 19 IDH1-R132H negative tumours (16%) were located in the frontal lobe. The average tumour volume was 50.1 ± 42.4 cm^3^. Although IDH1-R132H positive tumours tended to be larger than IDH1-R132H negative tumours, the difference was not statistically significant (62.2 ± 50.0 cm^3^ vs. 37.9 ± 29.6; p = NS). Similarly, there was no difference in the volume of contrast-enhancing tumour between IDH1-R132H positive and IDH1-R132H negative tumours (10.6 ± 23.9 cm^3^ vs. 12.1 ± 24.3 cm^3^; p = NS) and an equal percentage (33%) of IDH1-R132H positive and IDH1-R132H negative tumours demonstrated at least 5 cm^3^ of contrast-enhancement. Relative to IDH1-R132H negative tumours, IDH1-R132H positive tumours demonstrated a significantly higher minimum ADC (1.115 x 10^-3^ ± 0.326 x 10^3^ mm^2^/sec vs. 0.838 x 10^-3^ ± 0.266 x 10^3^ mm^2^/sec; p = 0.016) (Fig 1C and 1D).

A ROC analysis was performed to determine if any of the MRI features assessed could be used to predict the IDH1-R132H status of the tumour. This analysis demonstrated that of all the features assessed, only frontal lobe tumour location (sensitivity = 72.2%, specificity = 63.3%; AUC = 0.735; 95% CI = 0.571–0.898; p = 0.016) and minimum ADC (optimal cut-point ≥ 0.950 x 10^-3^ mm^2^/sec; sensitivity = 76.9%, specificity = 65.2%; AUC = 0.711; 95% CI = 0.534–0.887; p = 0.033) demonstrated moderate predictive power; none of the other features had a AUC ≥ 0.700.

### Association between markers of tumour cell proliferation in IDH1-R132H mutation status

Cell proliferation was quantified in all tumours using two measures: mitotic rate and Ki-67 LI. Compared to IDH1-R132H negative tumours, IDH1-R132H positive tumours demonstrated a significantly lower Ki67 LI (0.14 ± 0.10 vs. 0.24 ± 0.15; p = 0.034). In contrast, there was no difference in the mitotic index between IDH1-R132H positive and IDH1-R132H negative tumours (6.9 ± 7.0 /mm^2^ vs. 13.0 ± 22.5 /mm^2^; p = NS). However, after ROC analysis, neither Ki67 (AUC = 0.688; p = NS) nor mitotic index (AUC = 0.569; p = NS) demonstrated significant predictive power for the IDH1-R132H status of the tumour.

### Radiological and pathological variables associated with early mortality according to IDH1-R132H mutation status

Next we sought to identify which MRI features or markers of cellular proliferation were related to the risk of early mortality. Moreover, we hypothesized that the factors would differ by IDH1-R132H mutation status. The summary of this analysis is shown in Table 3. Among patients with IDH1-R132H positive tumours, age ≥ 50 years and volume of CE tumour ≥ 5 cm^3^ were associated with death within 12 months (Fig 1E and 1F). In contrast, in patients with IDH1-R132H negative tumours, only edema ≥ 1 cm beyond the tumour margin and mitoses < 5 / mm^2^ were positively associated with an increased risk of death. No other features were significantly associated with the risk of death at 12 months.

**Table 3:** Association between radiological features, markers of cellular proliferation, and early mortality in patients with newly diagnosed anaplastic astrocytic tumours.

	IDH1-R132H positive patients		IDH1-R132H negative patients	
	Number of events	p-value	Number of events	p-value
*Tumour location*		NS		NS
Frontal lobe	2/10		1/3	
Non-frontal lobe	1/8		8/15	
*Degree of enhancement*		NS		NS
Less than choroid plexus	0/8		2/7	
Equal to or greater than choroidplexus	3/10		7/11	
*Heterogeneity of enhancement*		NS		NS
None/homogeneous	1/7		2/4	
Heterogeneous	2/11		7/14	
*Volume of contrast-enhancing tumour*		**0.007**		NS
< 5 cm^3^	0/12		6/12	
≥ 5 cm^3^	3/6		3/6	
Peri-tumoural edema		NS		**0.046**
< 1 cm beyond tumour	1/13		4/12	
≥ 1 cm beyond tumour margin	2/5		5/6	
*Mass effect*		NS		NS
None/mild	2/12		4/13	
Moderate/severe	1/6		5/6	
*Intra-tumoural hemorrhage*		NS		NS
Present	0/2		1/2	
Absent	3/16		8/16	
*Cyst formation*		NS		NS
Present	0/3		0/1	
Absent	3/15		9/17	
*Tumour margin*		NS		NS
Irregular	2/8		5/8	
Smooth	1/10		4/10	
*Min apparent diffusion coefficient*		NS		NS
< 0.950 x 10^-3^ mm^2^/sec	2/5		4/11	
≥ 0.950 x 10^-3^ mm^2^/sec	1/13		5/7	
*Mitoses*		NS		**0.009**
< 5 / mm^2^	1/11		8/11	
≥ 5 / mm^2^	2/7		1/8	
*Ki67 labelling index*		NS		NS
< 10	0/8		3/4	
≥ 10	3/10		6/15	

Events are deaths occurring within 12 months of the original diagnosis.

Abbreviations: NS = not significant.

## Discussion

Patients presenting with diffuse anaplastic astrocytic tumours, including AAs and AOAs pose a diagnostic and clinical dilemma. Up to half will harbour a mutation in IDH1 which suggests that they arose from a previously undiagnosed low-grade tumour. On the contrary, IDH1-negative tumours may represent an 'early' glioblastoma or, indeed, a bona fide glioblastoma which has been under-sampled. The results of the present study demonstrate that despite their biological differences, anaplastic astrocytic tumours with and without the IDH1-R132H mutation share many radiological and pathological features with a few notable exceptions.

Patient age is the demographic parameter most robustly associated with IDH1 mutation status and also a strong predictor of outcome. Indeed the results of the present study are consistent with previous studies which found that the vast majority of patients diagnosed with IDH1-positive gliomas are younger than 50 years old. On the other hand, IDH1-positive gliomas are relatively rare in the paediatric population. Indeed, Lai and colleagues found that the probability of a tumour harbouring the IDH1 mutation abruptly increases at 20 years of age only to decrease again later in life.[9] One possible explanation is that the plasticity of the adolescence cortex and the activity of neural precursors provides a fertile ground for the development of tumours through an IDH1 dependent pathway. In contrast, astrocytic tumours arising in older adults may arise from a glial or multi-potential stem cell that normally resides in the subventricular zone and becomes neoplastic through an alternative cell signalling pathway. Indeed, a recent study demonstrated that cells harbouring both a gain of chromosome 7 and a loss of chromosome 10 are the likely cells of origin for primary glioblastomas.[10] In particular, a gain of chromosome 7 results in the amplification of EGFR which is commonly over expressed in IDH1-negative tumours. Subsequent mutations in the tumour suppressor genes PTEN and p53 then appear to drive the development of IDH1-negative tumours.[3] However, as our results show, IDH1-R132H negative tumours can occur in young adults just as IDH1-R132H positive tumours can occur in older adults. These uncommon cases are informative in the context of disentangling the relative contributions of IDH1 mutation status and age as older adults do poorly despite IDH1 mutation positivity and favourable pathology.

In addition to patient age, IDH1 status may be predicted from the imaging features of the tumour. In a study looking at low-grade gliomas, Metellus and colleagues demonstrated that tumours harbouring the IDH1 mutation are smaller and less likely to have an infiltrative pattern on MRI.[6] Similarly, Carrillo and colleagues found that IDH1-positive glioblastomas were non-CE and were more likely to be located in the frontal lobe.[5] Recently, MR spectroscopy has been used to identify mutant tumours through the non-invasive detection of the 2-hydroxyglutarate.[11, 12] In the present study we also found that the majority of IDH1-R132H positive tumours (55%) were located in the frontal lobe. The presence of the IDH1-mutation in most low grade gliomas but relatively few primary glioblastomas and the predilection for the frontal lobe raises the possibility that the tumour progenitors susceptible to IDH1 mutation normally reside in this region or migrate there prior to tumourigenesis.[9] In contrast to the previously described study, one-third of the IDH1-R132H positive tumours in the present study demonstrated large areas of avid CE. This discrepancy may be explained by the different methods used to define a tumour as 'non-CE' or by the small number of IDH1-positive tumours in the previous study. At the very least, our results demonstrate that the presence of CE on MRI does not rule out an IDH1-positive tumour. Another novel finding of the present study is that IDH1-R132H positive tumours have a higher minimum ADC (optimal cut-point ≥ 0.950 x 10^-3^ mm^2^/sec) when compared to IDH1-R132H negative tumours although this feature only had moderate power for predicting the status of the tumour. The ADC measures the impedance of water molecule diffusion; water in a tissue with a lower ADC is freer to move. Previous studies have shown that a low minimum ADC correlates with areas of high cellularity in gliomas. Indeed, Kitis and colleagues demonstrated that the minimum ADC could be used to differentiate between low-grade and high-grade gliomas although they did not find a difference between anaplastic gliomas and glioblastomas.[13] In another report, Murakami and colleagues found that in low-grade tumours, regions demonstrating the lowest ADC corresponded to high-grade foci.[14] Finally, Faguer and colleagues found that glioblastomas imaged early in their development tended to have a low ADC.[15] Taken together, these results suggest that IDH1-negative anaplastic astrocytic tumours demonstrate higher cellularity which is a feature associated with glioblastoma while IDH1-positive tumours continue to demonstrate features consistent with their low-grade precursors.

Isocitrate dehydrogenase is an enzyme which participates in the citric acid cycle where it catalyzes the oxidative decarboxylation of isocitrate to alpha-ketoglutarate and reduces NAD(P)+ to NAD(P)H. In humans, IDH1 and IDH2 perform similar functions and share considerable sequence similarity.[16] A mutation in either IDH1 or IDH2 has been shown to be a very early event in the development of low-grade gliomas. A mutation in either gene confers upon the mutant enzyme a neo-function, resulting in the production of the onco-metabolite D-2-hydroxyglutarate (D2H) which through genome-wide histone and DNA modification results in a hypermethylated phenotype.[17–19] Paradoxically, after transformation, D2H may actually restrain tumour growth. For example, *in vitro* studies have shown that the accumulation of DH2 in tumour cells decreases cell proliferation through inhibition of the cell cycle and depletion of metabolic substrates.[20, 21] Indeed, in cells expressing the IDH1 mutation, cell proliferation decreased in a dose dependent manner as the concentration D2H increased and this effect was associated with decreased activity of AKT, an enzyme linked to several cell survival pathways.[22] In the present study we show for the first time that IDH1-R132H positive tumours have a lower rate of proliferation as measured by expression of the cell cycle antigen Ki67. This result could explain why IDH1-positive tumours develop over years as opposed to months and why IDH1-positive tumours take longer to recur after resection. Taken together, these results suggest that while mutation of the IDH1 gene may initially drive tumourigenesis, it actually slows tumour growth by reducing cell proliferation. By the time IDH1-positive tumours transform into glioblastoma, however, they may have acquired additional mutations that allow them to increase their proliferative capacity.[9]

Previous studies have demonstrated that the IDH1 mutation is a favourable prognostic factor in patients with high grade gliomas.[8, 23–26] Indeed, a novel scoring system has recently been devised using IDH1 and additional molecular markers to predict prognosis for patients with anaplastic gliomas.[27] Similarly, IDH1 mutation and MGMT methylation status have been shown to predict survival in patients with AA treated with chemoradiation.[28] In the present study we demonstrate that even among IDH1-R132H positive patients, specific radiological and pathological features can be used to identify patients at greater risk for death within the first 12 months and that these features differ in patients with IDH1-R132H negative tumours. First, we found that patients with IDH1-R132H positive tumours and a volume of CE greater than 5 cm^3^ were more likely to die within the first 12 months compared to patients with IDH1-R132H positive tumours and less CE. Moreover, this association was not observed in patients with IDH1-R132H negative tumours where death occurred in patients with even minimal CE. This result is interesting in the context of a recent study by Beiko and colleagues which found that complete resection of CE tumour was associated with a survival advantage in patients with IDH1-positive but not IDH1-negative type tumours.[29] Thus, in IDH1-positive tumours, CE may represent a focus of transformation that is both prognostic and represents a target for surgical resection. Second, we found that in patients with IDH1-R132H negative tumours, edema extending more than 1 cm beyond the margin of the tumour was associated with a higher rate of death at 12 months. Anaplastic astrocytic tumours are a heterogeneous group of tumours and the degree of microvascular proliferation can vary greatly between tumours. These new vessels are inherently 'leaky' which results allows fluid move from the blood into the brain. Accordingly, the poor prognosis associated with increasing amount of peri-tumoural edema supports the need to reduce microvascular proliferation when treating high-grade gliomas.[30] Third, we found that in patients with IDH1-R132H negative tumours, a mitotic index greater than 5 / mm^2^ was associated with a lower rate of death at 12 months. The factors accounting for this frankly counter-intuitive finding remain to be elucidated. However, both chemotherapy and radiation therapy have been shown to be most effective against highly proliferative tumours and almost all the patients in this study received one or both modalities.

The cases presented in this report were diagnosed according to the 2007 WHO classification of CNS tumours.[2] According to that system, a diffusively infiltrative astrocytic tumour with anaplasia and mitotic activity is designated as a grade 3 tumour (anaplastic astrocytoma). Tumours that meet these criteria but also possess an oligodendroglial component are designated as anaplastic oligoastrocytomas. However, a recent consensus conference has recommended that the next WHO classification system incorporate molecular data so that diagnoses represent an integration of histologic and molecular information.[31] According to the proposed system, tumours with an 'oligoastrocytic' morphology but lacking the a deletion for 1p/19q would still be designated as astrocytoma. Indeed, the diagnostic entity 'oligoastrocytoma' has been removed from the proposed classification system. As such, all of the tumours diagnosed as oligoastrocytomas in our study would be re-classified as astrocytomas should this new system be adopted.

This study has several limitations. First, it is a relatively small study. This is partially a consequence of the fact that patients presenting with newly diagnosed anaplastic gliomas represent a minority of all patients diagnosed with primary brain tumours. As such, few studies have focused on the pathological and radiological properties of patients with these tumours. However, despite the small number of patients in this study, we were able to detect statically significant differences in several important variables and we believe these results would be reproducible in a larger validation cohort. The size of the study also limited the types of statistical analyses that could be performed. For example, it was inappropriate to perform a multivariate analysis to identify variables independently associated with death because several groups were characterized by ‘zero’ events (e.g. no patients less than 50 years old and with IDH1-R132H positive tumours died). Accordingly, these results would need to be confirmed in larger, prospective studies. Second, we do not present long-term follow-up data for the 67% of patients who survived beyond the primary endpoint (12 months after the date of diagnosis). However, the purpose of this study was to identify variables that could be used to distinguish between IDH-R132H positive and IDH-R132H negative tumours and identify variables associated with early mortality; accordingly, the study was designed with that purpose in mind. Third, IDH1 mutant tumours were identified by immunohistochemical analysis using an antibody that is specific for the R132H mutation. While this mutation is responsible for approximately 90% of all IDH1 mutant tumours, our study may have underestimated the actual number of IDH1-positive tumours by as much as 10%.[4] Finally, biopsy can result in under sampling and 12 patients in our study underwent biopsy-only prior to treatment. Of these, two patients with IDH1-R132H negative tumours demonstrated ring-enhancing lesions on their pre-operative MRI; a pattern more consistent with a glioblastoma. Although neither patient demonstrated radiological or pathological progression to glioblastoma during the over the 12 months of follow-up, we cannot rule out the possibility that the biopsy in these two cases was not fully reflective of the lesion.

The results of the present study may have important clinical implications. First, radiological data may be exploited by clinicians to better predict the IDH1 status of the tumour, in particular cases when a tissue diagnosis is not possible. Second, the results can also be used by clinicians and pathologists to identify patients at increased risk for early death, which might alter clinical decision making or discussions of prognosis with the patient.
